# Population size estimation of female sex workers using network scale-up method in Kermanshah city

**DOI:** 10.1186/s12889-023-15141-9

**Published:** 2023-02-07

**Authors:** Lida Olfati, Roya Safari-Faramani, Farid Najafi, Mehdi Moradi Nazar, Ebrahim Shakiba

**Affiliations:** 1 Student Research Committee, School of Health, Kermanshah University of Medical Sciences, Kermanshah, Iran; 2 Department of Epidemiology, School of Health Research Centre for Environmental Determinants of Health Research Institute for Health, Kermanshah University of Medical Sciences, Kermanshah, Iran; 3 Department of Biochemistry, School of Medicine Social Development and Health Promotion Research Centre Research Institute for Health Kermanshah, Kermanshah University of Medical Sciences, Kermanshah, Iran

**Keywords:** Female sex workers (FSWs), Network scale-up, Population size estimation

## Abstract

**Background:**

Appropriate estimate of size of the female sex workers as well as the other hard-to-reach-populations plays a crucial role in reaching them with effective preventive measures. This study aimed to estimate the population size of female sex workers in Kermanshah City using the network scale-up method.

**Method:**

In this cross-sectional study, of the 1000 pedestrians aged between 18 and 65 years, selected from the population of Kermanshah City via a multistage sampling, were recruited in the study. Face-to-face interview using a questionnaire including the number of FSWs in the social network of the respondent was used to collect the data.

**Results:**

The overall estimated number of female sex workers in the general population was 4848(UI 95%: 4597, 5074). Accordingly, the prevalence of FSWs among women 18 years and older in Kermanshah in 2018 was about 11.92 (UI 95%: 11.30, 12.47) in one thousand. More than half of the female sex workers in the respondent’s social network were unmarried and were in the age range of 18 to 29, and had a high school or university degree. Among high-risk behaviors, a history of alcohol consumption accounted for the highest percentage.

**Conclusion:**

The number of female sex workers estimated in this study is considerable, which highlights the importance of planning harm reduction interventions in order to alleviate the burden of HIV infection in the population.

## Introduction

Female Sex Workers (FSW) are one of the key hard-to-reach population and play a crucial role in the epidemiology of sexually transmitted infections (STIs) such as Human Immunodeficiency Virus (HIV). Comparing to the general population of women of reproductive age they disproportionately shoulder the more burden of HIV infection [1]. Although HIV epidemic in Iran has been concentrated in people who inject drugs (PWID), sexual transmission of HIV is on the rise [2, 3]. According to the latest evidence, prevalence of HIV in FSWs has **been** reported as high as 2.1% in 2015 [4]. In addition, both mortality and Disability-adjusted life years attributed to Acquired immunodeficiency syndrome (AIDS) has been increase during the last decades in Iran as well as other countries of MENA [5].

Appropriate estimate on the size of this population is important not only to understand the epidemiology of HIV and other STIs but also it is extremely important in planning effective preventive measures. Which is challenging because of marginalized nature of this population [6]. Particularly in Iran where due to cultural and religious values, this population encounter a range of challenges [7]. Therefore, use of direct method of sampling such as census, enumeration or household survey may results in underestimation if feasible because of the need to a large sample size as a result of low prevalence as well as practical difficulties [8]. Ample of evidences are available on size estimation of hard-to-reach population such as PWID, men who have sex with men (MSM), as well as FSWs via using indirect methods such as mapping, multiplier method, capture-recapture, and network scale up method (NSUM) [8].

NSUM primarily introduced in 1985 by an anthropologist, recently adapted to estimate the size of hard-to-reach populations [9]. NSUM comparing to the other methods has a great advantage. It relies on the information about the behavior of the respondent’s acquaintances whom are unknown to the interviewer and this prevent disclosing a known individual’s behavior [8]. It is widely used across the world such as Japan, Georgia, Singapore, Thailand, Brazil, and China to estimate the population of hard-to-reach population such as PWID, MSM FSW [10–16]. In Iran during the recent decades, it has been used to estimate population size in a wide range of sensitive topics such as abortion, alcohol and drug use, methadone maintenance therapy failure ratio, marijuana consumer, cancer, disability, and hard-to-reach-populations at both national and sub-national [8, 17–33].

Given the importance of providing updated information on the number and the size of population at risk of HIV as well as the other types of STIs to inform public health policy makers, this study aimed to apply NSUM in Kermanshah city to estimate the size of FSWs in 2018.

## Method

This is a cross-sectional study conducted between October 2018 and January 2019 in Kermanshah city using the network scale-up method. Kermanshah is the capital of Kermanshah Province, located in the westernmost part of Iran, with a population standing at one million people. Most of the population of the Kermanshah speaks Kurdish and it is the largest Kurdish-speaking city in Iran.

## Data collection

Regarding to the sensitivity of the issue in the context of Iran, previous researches underscore that more valid responses would be provide via using street-based interviews. So the data collection was based on the face to face interview with a gender matched interviewer and a structured questionnaire. In addition, baseline characteristics including age, education, marital status and occupation of the participants and data on age groups, education level, marital status and number of children of the known FSWs in the social network of the participants were collected. Eligible subjects were asked about the number of female sex workers that they know among their friends, relatives, colleagues, and neighbors or if they were married among their spouse’s. In this study “female sex workers” are defined as “women who have had sexual contact (vaginal, oral or anal) with a man in exchange for money or some kind of service. (such as providing food or a place to sleep)”. To know someone means the subject recognizes the person by their face or their name has seen them in person at least once in the past two years or has been in touch with them via email or cell phone. They are also able to contact each other, using the mentioned methods whenever they want.

## Sample size calculation

The sample size estimation was based on a reported prevalence of 0.015 by Sharifi, et al. [24], a sampling error of 0.008 and a 95% confidence level and 10% nonresponse.

## Study participants

In this study, 495 men and 505 women aged 18 and 65 years’ old who had lived in the city of Kermanshah for at least last 5 years were recruited following a multistage sampling approach. Stages were as follow: at first the city divided in to eight districts based on eight municipal districts. In each districts the required sample was determined proportion to the size of the districts. According to the age and sex distribution of the latest census in the general population, on average around 63 women and 62 men were selected in each district. In the second stage in each district, neighbors were categorized according to the high, middle and poor socioeconomic status. Since there were no available formal information on the socioeconomic status of the neighbors the expert’s opinions whom were the governmental bodies, working in the health centers of the city was used. In this stage 111 neighbors were selected across all districts of the city. The number of neighbors of high, middle and low socioeconomic status were 34, 48, and 29, respectively. In each neighbor’s busy streets, parks, and malls were selected to interview with the participants. Participants who were desired with informed consent to participate in the study and gave informed verbal consent were recruited.

Data collection was done from October 6th to December 15th, 2018. The schedule for interviewing the pedestrians has been set every day except Friday (an official holiday in Iran) in the morning from 9 to 12 and afternoon from 4 to 7. Interviewers were gender matched and female interviewers only interviewed women and male interviewers only interviewed men. Pedestrians on busy roads were randomly selected to participate in the study. After the description of the study, if they desired to participate in the study, they entered the study.

## Network scale up method

Network scale up method is one of the common method of size estimation, based on the collected data of the general population. In this method respond on behalf of their network rather than themselves. It assumes that the prevalence of a behavior in the network of the participants can be generalized to the whole population.

The estimation of the number of female sex workers was calculated using the formula below:

$$e=\frac{{\varSigma }_{i}{m}_{i}}{{\varSigma }_{i}{c}_{i}}\times t$$

$e$: the estimated number of female sex workers in the study

${\varSigma }_{i}{m}_{i}$: Sum of the number of female sex workers the participant *i* know in her/his social network.

${\varSigma }_{i}{c}_{i}$: the size of the personal network size (the number of people the participant knows by the definition)

In the present study, the personal network size for men and women participant was considered as 90 and 120, respectively and shows that on average, each man (woman) knows 90 (120) women in the reproductive age [28, 34].

*t*: The total population of women (≥ 18 years old) in Kermanshah city, based on the official 2016 Iran census, which equals 406,916.

*Visibility factor (VF)*: regarding the stigmatized nature of sex work, participants may not know about a person’s behavior in their social network. This may result in underestimation of the population of female sex workers that is known as *transparency barrier bias*. To deal with the crude estimates have been multiplied to =2.22 [35].

*Popularity factor (PF)*: T One of the main assumption of NSUM is that the network size of the members of the hidden and general populations are the same. Which may violated again due to stigmatized nature of the behavior. To take into account this crude estimates were multiplied by [35].

The uncertainty level for estimations was approximated using the Monte Carlo simulation, assuming a Poisson distribution for “m” (with mean equal to that of the responses) and “${c}_{i}$”. Also a uniform distribution for “VF” (in the range of $\frac{1}{0.55}=1.82$ to $\frac{1}{0.35}=2.86$ ) and “PF” (in the range of $\frac{1}{0.87}=1.15$ to$\frac{1}{0.67}=1.49$).

## Sensitivity analysis

As the network size was borrowed from another study two sensitivity analyses conducted one by assuming the network size in Kermanshah is 10% higher, and one by assuming the network size in Kermanshah is 10% lower the network size in Kerman.

## Ethical approval

The study protocol of the present study was approved at the Ethics Committee of Kermanshah University of Medical Sciences, under No. IR. KUMS.1397.416.

## Results

Of the 1390 approached eligible participants, 1000 were included in the study. Almost half of the participants were female (50.5%). Around 40% were aged 30 and 44. About two-thirds of the participants had either a high school diploma or a university degree and were married (Table 1).

**Table 1:** Demographic characteristics of the participants

Variable	Category	Frequency	%
gender	Male	495	49.5
	female	505	50.5
Age groups	18–29	315	31.4
	30–44	387	38.4
	45–65	299	29.9
Education level	Illiterate	62	6.2
	Elementary	91	9.1
	Guidance	191	19.1
	Diploma	656	65.6
Marital status	Single	313	31.3
	Married	669	67.3
	Divorced/Widow	18	1.6

The crude number of female sex workers in the social network of the general population was 434. Most of the FSWs were aged 18 and 29 and around half of them were educated at guidance level. Around 40% were divorced or widow and 56% have not any children. Demographic characteristics of FSWs in the general population’s social network is presented in the Table 2.

**Table 2:** Demographic characteristics of female sex workers in the participant’s social network

Variable	Category	Frequency	%
Age groups	18–29	259	59.6
	30–44	155	34.1
	45–65	20	4
Education level	Illiterate	11	2.5
	Elementary	46	10.5
	Guidance	206	47.4
	Diploma & higher	171	39.4
Marital status	Single	145	33.4
	Married	121	27.8
	Divorced	132	30.4
	Widow	36	8.2
Number of children	No child	243	55.9
	One	101	23.2
	Two	71	16.3
	≥ 3	19	4.3
Life time History of alcohol use	Yes	126	29.1
Life time history of prison	Yes	22	5.1
Life time history of drug use	Yes	64	14.7

Population size estimation of female sex workers.

Finally, the population of female sex workers in the Kermanshah city was estimated to be 4848 in 2018 (95% Uncertainty Intervals [UIs] 4597, 5074). Based on this, the prevalence of sex work in adult women aged 18 and more in Kermanshah was about 1.04%, in 2018.

The number of FSWs ranges from 4406 to 5386 when assuming the network size in Kermanshah is 10% higher, and it is 10% lower than the network size in Kerman, respectively.

Crude estimate, adjusted for visibility popularity, and both factors were as follow respectively: 1680, 3733, 2182, and 4848. The estimated uncertainty levels based on the Monte Carlo estimation and Bootstrap is presented in Table 3.

**Table 3:** Crude estimate, and estimated adjusted for visibility, popularity, and both factors with bootstrap and Monte Carlo uncertainty levels of the number of FSWs in Kermanshah in 2018

Method of estimation	point estimation	Crude estimate	Estimate adjusted for visibility factor	Estimate adjusted for popularity factor	Estimate adjusted for visibility factor and popularity factor
		1680	3733	2182	4848
Monte Carlo	percentile 25	1627	3580	2077	4597
	percentile 75	1735	3882	2266	5074
Bootstrap	Lower limit	1465	3254	1902	4226
	Upper limit	1888	4194	2451	5447

## Discussion

Regarding the importance of local estimates to tailor health interventions in order to avert number of new HIV/STI cases, this study primarily designed to estimate size of the FSW population as one of the important hard to reach population. According to results of the study, there are more than 4000 women in Kermanshah city in 2018 had exchanged sex for money, goods, or favor in the previous year. Regarding the estimated prevalence of HIV among FSW population based on the national surveys, which is 2.1, the projected number of HIV positive FSW in urban area of Kermanshah need to be strongly backed by the appropriate health programs [4].

Based on the results of our study 1.2% adult women aged 18 and more in Kermanshah had exchanged sex for money goods or favor in the previous year, which is in line with figures reported in Iran and across the other parts of the world. Sharifi et al. reported a prevalence of 1 0.43 among urban Iranian women [24]. Fearon, et al. reported a prevalence of 1.23 in Zimbabwe [36] and Vandepitte et al. reported a prevalence around 0.2% and 2.6% in Asian countries [37]. Jing et al. reported the prevalence of FSW in women aged 15–49 at 1.3% [38].

Most of the FSWs in the social network of the participants were young, have been married at least once, with no children and educational level of lower than diploma. On the other hand, around one out of three and 15% have history of ever use of alcohol and drug use, respectively. Which are more or less similar to the results reported by previous studies based on the national data of National Bio behavioral Survey in 2010 and 2015 [4, 39].

Taking into account age-gender distribution, population density and socioeconomic status of the neighborhoods, we tried to select a representative sample of the general population. Also, the sampling was proportionate to the latest census. In the 1395 census, the highest share of the population belonged to 18–44 age groups, and the share of other age groups was similar to the samples taken in the study. Similar to this study, in the 1395 census, the highest population share in terms of education, belonged to holders of high school diplomas and higher. In the 1395 census, 60% of the population was married, 32% single and the rest widowed or divorced. Having concluded that the study participants were at most representation of the general population. On the other hand, this is one of largest studies of this size to our knowledge. Previous studies conducted in Kermanshah were based on smaller sample size.

Using NSUM provide opportunity to ask about the acquaintances. Although it is affected by the visibility of the behaviors in their social network. Like the other studies we tried to adjust the estimations by considering the correction factor for visibility based on the study conducted in Shiraz located in the south of Iran which was the best available and widely used across the studies [24, 35]. Cultural differences between these two cities may impose effect on the estimations. That merit further investigations to consider probable differences.

Another limitation of this study was that only one method was used for estimation. Due to the limitations posed by indirect methods, use of alternative methods in a single study is recommended [6]. Sharifi, et al. has used two methods other than NSUM including wisdom of the crowds and multiplier methods. The results yielded distinctly different results ranges from 72 based on the multiplier method and 4000 using NSUM [24]. In this study, access to more reliable sources than the previous studies were not feasible to apply. On the flip side, using other methods such as mapping and capture re-capture was not possible due to lack of independent sources of information and financial limitations. Another limitation was applying the social network size calculated in Kerman city. Although up to our knowledge evidences on evident differences in the case of social network across different parts of Iran is limited. That merit further investigation.

In this study, we asked participants about the number of prostitutes in their social networks. Vividly this needs time and a good memory to remember all the members of the social network. Due to the fact that people may have limitations in remembering to facilitate we asked about the social network member in different subgroups such as their own friends and the friends of their wife/husband if married, colleagues and the colleagues of their wife/husband if married and neighbors. Participants were given enough time to provide an accurate count. However, it always threatens some degree of limitation in recalling particularly about the complementary questions on marital status, education level as well as their risky behaviors. Therefore the results should be interpreted with caution.

Sexual behaviors are among the most sensitive issues in human societies. In this study we aimed to estimate the size of one of the stigmatized sub populations using an indirect method, NSUM. NSUM is widely used in Iran for a wide variety of issues and its results is to some extant reliable [23, 24]. Regarding the sensitivity of the issue, the related stigma and other challenges estimating a precise number is far from simple. Repetition of the results across the studies conducted in Kermanshah city by others [24] is a good indication of the reliability of the method results although due to lake of gold standard the results must be interpreted with caution.

## Conclusion

In this study, the prevalence of sex work in the population of women, aged 18 and older, in Kermanshah city was estimated to be 1.2% which equals 4848 female sex workers. The difference between the results of this study and the recorded statistics in harm reduction centers, calls for more attention from officials to devise the means to effectively access female sex workers to reduce the consequences of spreading sexually transmitted diseases, especially HIV. Measures must also be taken to improve mental health services in society. In the harm reduction programs, more research needs to be conducted about the way female sex workers and other vulnerable groups dress. Studying correction multipliers, such as visibility factor and popularity factor, and social network size in different parts of the country with different cultural and social contexts can help increase the validity of estimations in the future.

## Data Availability

The datasets used and/or analysed during the current study are available from the corresponding author on reasonable request.
